# Rückgang der Arbeits- und Wegeunfälle während des 1. Lockdowns 2020 im Rahmen der SARS-CoV-2-Pandemie: Vergleich zu den Zeiträumen der Jahre 2015–2019

**DOI:** 10.1007/s00113-021-01023-5

**Published:** 2021-06-22

**Authors:** Kai Hoffeld, Patrick Pflüger, Dominik Pförringer, Martin Hofmeister, Fabian Stuby, Peter Biberthaler

**Affiliations:** 1 Klinik und Poliklinik für Unfallchirurgie, Klinikum rechts der Isar, Technische Universität München, Ismaninger Straße 22, 81675 München, Deutschland; 2 Berufsgenossenschaftliche Unfallklinik Murnau, Professor-Küntscher-Straße 8, Murnau, 82418 Deutschland

**Keywords:** Ressourcenallokation, Bewältigungsstragie, Straßenverkehrsunfallzahlen, VAV-/SAV-Verfahren, COVID-19, Resource reallocation, Coping strategy, Number of traffic accidents, VAV/SAV process, COVID-19

## Abstract

**Hintergrund:**

Zur Reduktion der SARS-CoV-2-Infektionszahlen wurden Maßnahmen wie z. B. Kontakt- und Ausgangsbeschränkungen getroffen, die schließlich im „1. Lockdown“ mündeten. Dies sollte Kapazitäten im Gesundheitssystem zur Bewältigung der Pandemie schaffen.

**Ziel der Arbeit:**

Es wird analysiert, ob während des Lockdowns die Anzahl der Arbeits- und Wegeunfälle im Vergleich zum Zeitraum der Jahre 2015–2019 gesunken ist.

**Material und Methoden:**

Es wurden retrospektiv alle Arbeits- und Wegeunfälle, die während des Beobachtungszeitraumes 16.03.–04.05.2020 im Klinikum rechts der Isar sowie in der Berufsgenossenschaftlichen Unfallklinik Murnau behandelt wurden, mit den Zahlen aus demselben Beobachtungszeitraum der Jahre 2015 bis 2019 verglichen. Die Daten über die Fallzahlen nach dem Durchgangsarztverfahren (DAV) wurden weitergehend nach dem Verletzungsartenverzeichnis der Deutschen Gesetzlichen Unfallversicherung (DGUV) in Fälle des Verletzungsartenverfahrens (VAV) und Schwerstverletzungsartenverfahrens (SAV) aufgeschlüsselt. Zudem wurden die erhobenen Daten mit Daten des Statistischen Bundesamtes zu den Zahlen der Verkehrsunfälle und Verkehrstoten verglichen.

**Ergebnisse:**

Es wurden insgesamt 4313 Fälle berücksichtigt. Im Jahr 2020 sank die Zahl der Arbeits- und Wegeunfälle im Vergleich zum Beobachtungszeitraum 2015–2019 um 31 %. Die VAV-Fälle waren 2020 um 26 % verringert. Die SAV-Fälle sind im Jahr 2020 um 5 % gesunken. Die Zahl der Straßenverkehrsunfälle sank in den ersten 4 Monaten im Jahr 2020 im Vergleich zu den ersten 4 Monaten 2019 um 17 %; die Zahl der Verkehrstoten sank um 11 %.

**Diskussion:**

Es zeigt sich ein Rückgang der Arbeits- und Wegeunfälle um 31 %. Dies hat zur Ressourcenreallokation im Rahmen der Pandemie beigetragen. Dennoch zeigen sich nahezu konstante Zahlen schwerstverletzter Patienten und Unfalltoter, was die Relevanz unfallchirurgischer Strukturen besonders in Krisenzeiten aufzeigt und in der Kalkulation der intensivmedizinischen Ressourcen unabdingbar macht.

## Abstract (en)

**Background:**

Measures such as restrictions on personal contact and going out were taken to reduce SARS-CoV‑2 infection numbers, ultimately resulting in the first lockdown. This was intended to create capacity in the healthcare system to manage the pandemic.

**Aim of the work:**

To analyze whether the number of work and commuting injuries decreased during the lockdown compared to the years 2015–2019.

**Material and methods:**

All work and commuting accidents treated at the Klinikum rechts der Isar and the Berufsgenossenschaftliche Unfallklinik Murnau during the observational period 16 March–4 May 2020 were retrospectively compared with the figures from the same observation period in the years 2015–2019. The data on case numbers according to the transit physician procedure (DAV) were further broken down into cases of the injury type procedure (VAV) and severe injury type procedure (SAV) according to the injury type index of the German Social Accident Insurance (DGUV). In addition, the collected data were compared with data from the Federal Statistical Office on the numbers of traffic accidents and traffic fatalities.

**Results:**

A total of 4313 cases were considered. In 2020, the number of work and commuting accidents decreased by 31% compared to the observation period 2015–2019. VAV cases were decreased by 26% in 2020. SAV cases decreased by 5% in 2020. Road traffic accidents decreased by 17% in thre first four months in 2020 compared to the first four months in 2019. Traffic fatalities decreased by 11%.

**Discussion:**

There was a 31% decrease in work and commuting accidents. This contributed to resource reallocation in the context of the pandemic. Nevertheless, almost constant numbers of severely injured patients and accidental deaths occurred, which shows the relevance of trauma surgery structures especially in times of crisis and makes it indispensable to include them in the calculation of intensive care resources.

## Einleitung

Das neuartige Virus „severe acute respiratory syndrome coronavirus 2“ (SARS-CoV-2) ist das auslösende Pathogen hinter der SARS-CoV-2-Pandemie [1, 2], die spätestens seit dem Frühjahr 2020 die ganze Welt vor neue Herausforderungen stellt. Zum ersten Mal wurde im Dezember 2019 in Wuhan in der Provinz Hubei in China die neue Atemwegserkrankung beobachtet. Daraufhin konnte am 07.01.2020 SARS-CoV‑2 erfolgreich isoliert werden [3]. Während am 30.01.2020 7734 Fälle von SARS-CoV-2-Infektionen in China und 90 Infektionen im Rest der Welt gezählt wurden, stieg diese Zahl innerhalb kürzester Zeit bis zum 16.02.2020 auf 51.857 in 25 verschiedenen Ländern weltweit an [4]. Die WHO (Weltgesundheitsorganisation) rief schließlich den Pandemiefall aus. Diese dramatische Entwicklung veranlasste viele Regierungen weltweit zu verschiedenen Maßnahmen, um die Ausbreitung des Virus einzudämmen [5]. Auch in Deutschland wurden zahlreiche Maßnahmen getroffen, die mit dem Absagen von Großveranstaltungen, Schließungen von Kitas und Schulen, Produktionsstopps in größeren Betrieben sowie Schließung des Einzelhandels und Verhängen von Ausgangsbeschränkungen schließlich im „Lockdown“ mündeten. So kam das öffentliche Leben größtenteils zum Erliegen. Nicht zuletzt durch großzügige Umstellung auf „Home-Office“-Tätigkeiten in vielen Betrieben zeigte sich auch eine massive Reduktion des öffentlichen Verkehrsaufkommens, was sich beispielsweise in der geringsten Zahl von Verkehrsunfällen innerhalb eines Monats seit der Wiedervereinigung 1990 niederschlägt [6]. Es wird untersucht, welche Auswirkungen die Kontakt- und Ausgangsbeschränkungen im Rahmen des Lockdowns auf die Anzahl der Arbeits- und Wegeunfälle im Vergleich zum Vergleichszeitraum der Jahre 2015–2019 hatten.

## Material und Methoden

### Klinikdaten

Um ein möglichst repräsentatives Bild zu erhalten, wurden in dieser Studie die Daten zum einen der Berufsgenossenschaftlichen Unfallklinik Murnau als des typischen Vertreters einer überregionalen BG-Klinik in ruraler Umgebung und zum anderen der Klinik und Poliklinik für Unfallchirurgie des Klinikums rechts der Isar in München als des typischen Vertreters eines urbanen Traumazentrums eingeschlossen. Beide Kliniken sind überregionale Traumazentren und sind am Verletzungsartenverfahren (VAV) sowie am Schwerstverletztenartenverfahren (SAV) beteiligt [7]. Dementsprechend versorgen beide Kliniken Arbeitsunfälle mit Verletzungen jeden Schweregrades.

### Datenerfassung und Auswertung

Die Studie ist eine vergleichende, epidemiologische, retrospektive Studie. Die Daten sind retrospektiv für den Zeitraum vom 16.03.2020–04.05.2020 erfasst worden. Als Vergleichswert wurde derselbe Zeitraum vom 16.03.–04.05. für die Jahre 2015 bis 2019 herangezogen. Für den Vergleichszeitraum der Jahre 2015 bis 2019 wurden dann entsprechende Mittelwerte gebildet. Es wurden alle im jeweiligen Referenzzeitraum der Jahre 2015 bis 2020 nach dem BG-Verfahren behandelten Patientenfälle in dieser Studie berücksichtigt. Die eingeschlossenen Fälle der Jahre 2015–2018 wurden nach dem Verletzungsartenverzeichnis in VAV- und SAV-Fälle kategorisiert [8]. Die Fälle der Jahre 2019 und 2020 wurden nach der Version 2.0 des Verletzungsartenverzeichnis vom 01.07.2018 in VAV- und SAV-Fälle kategorisiert. Für diese Studien wurden die Abrechnungsdaten der beiden Kliniken analysiert. Die Einteilung in VAV- und SAV-Fälle findet routinemäßig im entsprechenden BG-Verfahren statt. Es handelt sich somit um eine Sekundäranalyse.

Um die Veränderungen der Anzahl an Verkehrsunfällen bzw. Verkehrstoten zu analysieren, haben wir die Daten des Statistischen Bundesamtes herangezogen [6, 9].

Die Analyse aller Daten erfolgte mittels RStudio Team (2019; RStudio: Integrated Development for R. RStudio, Inc., Boston, MA, USA, http://www.rstudio.com/).

## Ergebnisse

**Abb. 1:**
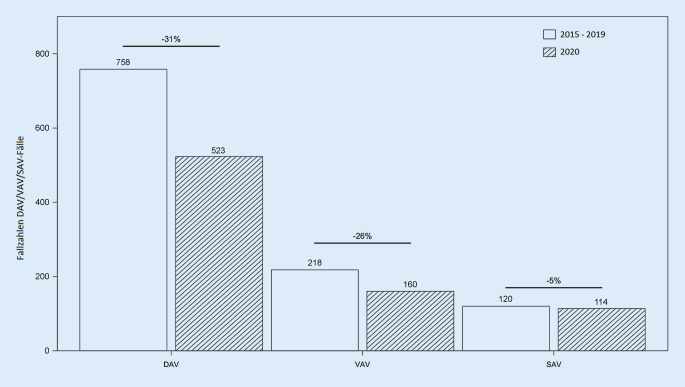
Fallzahlen für DAV, VAV- und SAV-Fälle für die verschiedenen Untersuchungszeiträume. Die prozentuale Angabe bezieht sich auf den Vergleich der Zahlen 2020 zu dem Mittelwert der Vergleichsjahre 2015–2019

Insgesamt sind 4313 Fälle für den Beobachtungszeitraum vom 16.03.–04.05. der Jahre 2015 bis 2020 berücksichtigt worden. Der Beobachtungszeitraum betrug jeweils 50 Tage. Insgesamt wurden in den Jahren 2015–2019 in den beiden analysierten Häusern im betreffenden Beobachtungszeitraum 3790 Arbeits- und Wegeunfälle behandelt, was einem Mittelwert von 758 Fällen entspricht. Im Jahr 2020 wurden im Beobachtungszeitraum mit insgesamt 523 behandelten Arbeits- und Wegeunfällen im Vergleich zum Mittelwert der Jahre 2015–2019 31 % weniger Unfallversicherte behandelt. Aufgeschlüsselt nach VAV- und SAV-Fällen zeigen sich folgende Werte. In den Jahren 2015–2019 wurden insgesamt 1088 VAV- und 597 SAV-Fälle behandelt. Somit ergeben sich die Mittelwerte von 218 behandelten VAV- sowie 120 behandelten SAV-Fällen. Im Jahr 2020 wurden in den beteiligten Kliniken insgesamt 160 VAV- und 114 SAV-Fälle behandelt. Bei den VAV-Fällen zeigt sich für beide Kliniken zusammengerechnet ein Rückgang um 26 % (Abb. 1). Die SAV-Fälle sind im Jahr 2020 im Vergleich zum Mittelwert der Jahre 2015–2019 um 5 % zurückgegangen. Die Zahl der stationär behandelten BG-Fälle sank im Vergleich zum Jahr 2019 um 59 %. Die Zahlen über die Verkehrsunfälle, Verkehrsverletzten und Verkehrstoten sind Tab. 1 zu entnehmen.

**Tab. 1:** Daten über Verkehrsunfälle. (Nach Daten des Statistischen Bundesamtes [6, 9])

Zeitraum	Verkehrsunfälle^a^	Verkehrsverletzte^a^	Verkehrstote
März 2019	204.000	25.900	234
März 2020	166.000	20.400	158
*Differenz*	*−23* *%*	*−27* *%*	*−32* *%*
April 2019	195.000	27.900	239
April 2020	144.500	21.000	236
*Differenz*	*−35* *%*	*−33* *%*	*−1* *%*
Q1 2019	824.900	82.300	880
Q1 2020	706.200	71.100	783
*Differenz*	*−17* *%*	*−16* *%*	*−11* *%*

^a^Auf 100 gerundet

## Diskussion

Die SARS-CoV-2-Pandemie hat in Deutschland gesamtgesellschaftliche und ökonomische Veränderungen ausgelöst. In der vorliegenden Studie konnten die Auswirkungen der veränderten Arbeitsstrukturen durch die Reduktion von Arbeits- und Wegeunfällen im Untersuchungsgebiet um 31 % nachgewiesen werden. Dies zeigt die Wirksamkeit der durch die Politik getroffenen Maßnahmen zur Ressourcenreallokation im Gesundheitssektor im Rahmen des Lockdowns während der SARS-CoV-2-Pandemie auf. Als Folge der reduzierten Fallzahl lässt sich eine Reduktion der stationär behandelten BG-Fälle um 59 % verzeichnen. Daraus ergaben sich freie Bettenkapazitäten, die für den Vollzug des Notfallplans zur Bekämpfung der Coronapandemie des Bayerischen Staatsministeriums des Inneren sowie für Gesundheit und Pflege vorzuhalten sind [10]. Neben dem Aussetzen von elektiven Operationen zur direkten Schaffung von Bettenkapazitäten wurden also durch den Lockdown und die dadurch geringeren Fälle von Arbeits- und Wegeunfällen indirekt zusätzlich freie Bettenkapazitäten geschaffen. Zu den VAV-Fällen zählen beispielsweise „komplexe Brüche der großen Röhrenknochen“ (Punkt 6, Verletzungsartenverzeichnis). Diese Verletzungen gehen meist mit einer Notwendigkeit einer Operation einher. Eine Reduktion von VAV-Fällen führt folglich zu einer Reduktion derartiger Operationen. Andere Arbeiten haben bereits gezeigt, dass durch die Reduktion von unfallchirurgischen und orthopädischen Operationen weniger (anästhesiologisches) Personal und weniger materielle Ressourcen (Beatmungsgeräte) gebunden werden, die somit für die Behandlung von Patienten mit einer SARS-CoV-2-Infektion zur Verfügung stehen [11]. Nichtsdestotrotz blieb die Anzahl der SAV-Fälle mit 95 % im Vergleich zum Mittelwert der Vergleichsjahre 2015–2019 nahezu gleich. SAV-Fälle benötigen jedoch häufig eine intensivmedizinische Behandlung, was die Kapazität der Intensivbetten während der SARS-CoV-2-Pandemie zusätzlich strapaziert. Anhand der weitgehend kontinuierlich hohen Zahlen der Schwerverletzten ist zu sehen, dass die Unfallchirurgie, unabhängig von den äußeren Rahmenbedingungen, einen konstant hohen Stellenwert hat und der Bedarf an Intensivkapazität auch in außergewöhnlichen Krisenzeiten berücksichtigt werden muss. Es zeigt sich also, dass die für die Bewältigung der Pandemie geforderte Ressourcenschaffung nur zu einem kleinen Teil durch Einsparungen in der Akutmedizin, aber v. a. durch Einschränkungen im Elektivbereich erreichen lässt.

Die Reduktion von Arbeits- und Wegeunfällen führt, volkswirtschaftlich betrachtet, zu monetären Einsparungen durch geringere Behandlungskosten aufgrund der geringeren Fallzahl. Die geringeren Behandlungszahlen haben jedoch auch Auswirkungen auf die jährlichen Budgetgespräche der betroffenen Abteilungen eines Klinikums. Die Anstrengungen zur Bekämpfung der SARS-CoV-2-Pandemie beinhalten u. a. das Aussetzen von elektiven Operationen und das Vorhalten von freien Bettenkapazitäten. Somit war das Jahr 2020 aus Abteilungssicht ein wirtschaftlich schwaches Jahr und wird sich dementsprechend negativ auf die Kalkulation der Budgetgespräche auswirken. Die tatsächlichen Leistungseinbußen durch die SARS-CoV-2-Pandemie lassen sich nicht zuverlässig beziffern, was die Zahlen als Grundlage für anfallende Budgetgespräche nicht verwertbar macht. Folgerichtig empfehlen bereits Giunta et al., dass die Zahlen aus dem Vorjahr 2019 für Verhandlungen und Anpassungen herangezogen werden [12]. Die vorliegenden Ergebnisse zeigen die beschriebene Problematik aus anderer Perspektive erneut auf und unterstützen diese Empfehlung.

Keinen Einfluss auf die Zahl der Arbeitsunfälle hatte die Tatsache, dass eine Erkrankung mit SARS-CoV‑2, auch bei beruflicher Exposition, zum Erhebungszeitraum nicht als Arbeitsunfall anerkannt war. Mittlerweile kann eine Infektion mit SARS-CoV‑2 infolge einer Beschäftigung außerhalb der Tätigkeitsbereiche im Gesundheitsdienst, in der Wohlfahrtspflege oder in einem Laboratorium einen Arbeitsunfall darstellen [13]. Dies bedarf in jedem Falle einer Einzelfallprüfung durch den zuständigen Träger. Entsprechend den offiziellen Stellungnahmen der Deutschen Gesetzlichen Unfallversicherung (DGUV) besteht allerdings die Möglichkeit zur Anzeige einer Berufserkrankung BK 3101 bei nachgewiesenem berufsbedingtem Kontakt und klinischer Symptomatik bzw. Erkrankung und positivem Virusnachweis, falls eine Beschäftigung in den oben genannten Tätigkeitsbereichen vorliegt [14]. Die Deutsche Gesetzliche Unfallversicherung teilte den Autoren dieser Studie auf Anfrage mit, dass zum Ende der 33. Kalenderwoche (16.08.2020) nach entsprechender Prüfung der Verdachtsmeldungen COVID-19 bislang 7509 Fälle von den Unfallversicherungsträgern als Berufskrankheit anerkannt worden sind. Die Infektionszahlen im Bereich des medizinischen Personals in Deutschland waren zu jenen in Spanien oder Italien aufgrund der unklar unterschiedlichen Datenerhebung nicht vergleichbar. Dennoch wäre ein Anstieg von Arbeitsunfällen durch die Erkrankung mit SARS-CoV‑2 zu erwarten gewesen. Dies ist, wie beschrieben, auf die ausbleibende Anerkennung als Arbeitsunfall zurückzuführen.

Die Staatengemeinschaft der Europäischen Union (EU) strebt auf vielen Ebenen eine stärkere Zusammenarbeit an. Der demografische Wandel, neue Gesundheitsbedrohungen, wie die aktuelle SARS-CoV-2-Pandemie, gesundheitliche und Versorgungsungleichheiten innerhalb einzelner sowie zwischen den Staaten der EU stellen die Gesundheitssysteme in Europa vor schwierige Aufgaben [15]. Um auf die Herausforderungen mit wirksamen gesundheitspolitischen Maßnahmen reagieren zu können, ist es sinnvoll, vergleichbare Gesundheitsdaten in Europa zu erheben. Sie können wertvolle Informationen über die Verteilung von Risiko- und Schutzfaktoren, Prävalenzen chronischer Erkrankungen und die Versorgungssituation in den Mitgliedstaaten liefern und so die Planung und Umsetzung der Maßnahmen unterstützen [15]. Die durch uns erhobenen Daten tragen zur Informationsgewinnung über die Wirksamkeit der getroffenen Maßnahmen im Rahmen der Eindämmung der SARS-CoV-2-Infektionen bei. Dadurch kann die Bewertung der Maßnahmen innerhalb Deutschlands und somit im europäischen Vergleich unterstützt werden und so dabei helfen, effektive Strategien zur Bewältigung der aktuellen sowie zukünftigen Pandemien zu erarbeiten. Besonders unter Beachtung des föderalistischen Systems in Deutschland und der unterschiedlichen Umsetzungen der verschiedenen Maßnahmenpläne durch die einzelnen Bundesländer könnte eine Ausweitung der Datenerhebung über die Veränderungen der Arbeits- und Wegeunfälle auf ganz Deutschland Rückschlüsse auf effektive und weniger effektive Maßnahmen liefern.

In einer kürzlich veröffentlichten Arbeit wurden die Effekte der SARS-CoV-2-Pandemie auf die Versorgung von Krankenhauspatienten bzw. die veränderte Versorgungsrealität untersucht. Dazu wurden die Abrechnungsdaten von 310 Kliniken im Beobachtungszeitraum 13.03.–19.04. für die Jahre 2019 und 2020 analysiert. Mit einem Rückgang auf durchschnittlich 57 % der Vorjahreszahlen zeigen die Daten eine Halbierung der Fallzahlen während des Lockdowns [16]. Die Reduktion der Fallzahlen im elektiven Sektor ist durch die erlassenen Maßnahmen zu erklären. Es kam darüber hinaus jedoch auch zu einer Reduktion von Notfallbehandlungen, wie beispielsweise Herzinfarkten, bei denen nur noch 66,1 % der Vorjahresfallzahlen zu verzeichnen waren [16]. Neben dem Erklärungsversuch, dass Patienten aus Angst vor einer Ansteckung die Notaufnahmen und andere Einrichtungen des Gesundheitswesens gemieden haben, beschreiben die Autoren die Möglichkeit, dass die Inzidenz von Notfällen aufgrund fehlender auslösender Faktoren von Herz‑, Kreislauf- oder Lungenerkrankungen, wie z. B. einer Infektion, durch die Kontaktbeschränkungen abnahm. Im Gegensatz dazu kommen unfallchirurgische Notfälle in der Studie auf 75,9 % (Schenkelhalsfrakturen) oder sogar 81,1 % (pertrochantäre Frakturen) der Fallzahlen aus dem Vorjahr [16]. Nach Pneumonien (95,6 %) und Geburten (91,6 %) ist die pertrochantäre Fraktur die einzige in der Studie analysierte Diagnose, die über 80 % der Fallzahlen des Vorjahres erreicht und somit nur einem unwesentlichen Einfluss durch die SARS-CoV-2-Pandemie unterliegt. In den ersten 4 Monaten im Jahr 2020 verzeichnet das Statistische Bundesamt insgesamt 706.200 Straßenverkehrsunfälle in Deutschland. Dies entspricht rund 17 % weniger Verkehrsunfällen als in den ersten 4 Monaten 2019. Betrachtet man die Monate des Lockdowns, so sind hier die Auswirkungen des pandemiebedingten geringeren Verkehrsaufkommens noch viel deutlicher zu beobachten. Im Monat März sanken Verkehrsunfälle 2020 um 23 % gegenüber der Anzahl der Verkehrsunfälle im März 2019, und im Monat April wurden sogar 35 % weniger Verkehrsunfälle im Jahresvergleich verzeichnet. Dieser Trend setzt sich bei den Verkehrsunfällen mit verletzten Personen fort. Im März 2020 sank die Zahl der Verletzten um 27 % und im April 2020 um 33 % [6, 9]. Die Zahl der Verletzten folgt also näherungsweise der Zahl der Verkehrsunfälle. Bemerkenswert ist jedoch die Veränderung bei den Verkehrstoten. Während im März 2020 noch eine Reduktion der Verkehrstoten um rund 32 % zu beobachten war, relativiert sich dies im April auf eine Differenz von nur 1,26 % weniger Verkehrstoten im Vergleich zum Vorjahr [6, 9]. Es zeigt sich also ein deutlicher Rückgang der insgesamt verzeichneten Straßenverkehrsunfälle und der insgesamt verzeichneten verletzten Personen bei Straßenverkehrsunfällen als Folge der getroffenen Eindämmungsmaßnahmen während des Lockdowns. Dennoch ist die Zahl der Verkehrstoten während des Lockdowns nur unwesentlich gesunken, was sich äquivalent auch in den nahezu unveränderten Zahlen der SAV-Fälle in den beteiligten Kliniken in unserer Studie sowie damit einhergehend in den weiterhin hohen Fallzahlen unfallchirurgischer Diagnosen in der Studie von Kuhlen et al. widerspiegelt.

## Limitierungen

Diese Studie hat die absolute Fallzahl der Arbeits- und Wegeunfälle in den jeweiligen Vergleichszeiträumen, die in den beiden Kliniken behandelt wurden, analysiert. Über die Zahl der Freizeitunfälle, die reziprok potenziell zugenommen hat, kann keine spezifische Aussage gemacht werden, da diese nicht erhoben wurden. Der Referenzzeitraum 2015–2018 ist nicht homogen klassifiziert, da sich der Verletzungsartenkatalog zum 01.07.2018 geändert hat. Die Mittelwertbildung der VAV- und SAV-Fälle für den Zeitraum 2015–2019 kann somit verzerrt sein.

## Fazit für die Praxis

Durch die veranlassten Maßnahmen wurde versucht die Ausbreitung der SARS-CoV‑2 Infektionen in Deutschland einzudämmen und zusätzliche Ressourcen im Gesundheitswesen zu schaffen. In der vorliegenden Studie wurde untersucht, wie sich diese Maßnahmen auf die Zahl der Arbeits- und Wegeunfälle und dadurch indirekt auf die zur Verfügung stehenden Ressorucen ausgewirkt haben. Es konnte für den Beobachtungszeitraum vom 16.03.–04.05.2020 im Vergleich zum Mittelwert des Referenzzeitraums 2015–2019 als wesentliche Veränderung die Reduktion von Arbeits- und Wegeunfällen um 31 % festgestellt werden, während die SAV-Fälle in den beteiligten Kliniken nahezu unverändert blieben. Weitere indirekte Auswirkungen, die durch diese Reduktion ausgelöst wurden, trugen zu einer Ressourcenreallokation im Gesundheitssektor während der Pandemie bei. Aus diesen Erkenntnissen lässt sich schließen, dass durch den Rückgang der Arbeits- und Wegeunfälle indirekt Ressorucen im Gesundheitswesen eingespart werden konnten. Diese Daten können zur Beurteilung einzelner Aspekte ger getroffenen Maßnahmen und zur Unterstützung der Planung zukünftiger Maßnahmen herangezogen werden. Die dennoch etwa gleich hohen Zahlen der SAV-Fälle sowie der Verkoehrstoten in Deutschland unterstreichen die Wichtigkeit der Aufrechterhaltung unfallchirurgischer Strukturen – insbesondere in Krisenzeiten. Ferner sollte bei der Kalkulation der Intensivbettenkapazität in vergleichbaren außerordentlichen Umständen, wie der SARS-CoV‑2-Pandemie, der intensivmedizinische Bedarf zur Versorgung schwerstverletzter Patienten mit einbezogen werden.

## Abkürzungen

DAV: Durchgangsarztverfahren
DGUV: Deutsche Gesetzliche Unfallversicherung
SAV: Schwerstverletzungsartenverfahren
VAV: Verletzungsartenverfahren
Verletzungsartenverzeichnis: Dieses Verzeichnis erläutert in 11 Ziffern die möglichen Verletzungen bzw. Verletzungsmuster und kategorisiert anhand der Verletzungsschwere diese in VAV und SAV
WHO: Weltgesundheitsorganisation
